# The contribution of a non-governmental organisation’s Community Based Tuberculosis Care Programme to case finding in Myanmar: trend over time

**DOI:** 10.1186/s40249-017-0253-y

**Published:** 2017-04-03

**Authors:** Htet Myet Win Maung, Saw Saw, Petros Isaakidis, Mohammed Khogali, Anthony Reid, Nguyen Binh Hoa, Ko Ko Zaw, Saw Thein, Si Thu Aung

**Affiliations:** 1 National Tuberculosis Programme, Ministry of Health and Sports, Zabuthiri township, Postcode 15011 Nay Pyi Taw, Myanmar; 2 Department of Medical Research, Ministry of Health and Sports, Yangon, Myanmar; 3 Médecins Sans Frontières, Operational Research Unit, MSF-Luxembourg, Luxembourg, Luxembourg; 4 National Tuberculosis Programme, Hanoi, Vietnam; 5 Centre for Operational Research, International Union Against Tuberculosis and Lung Disease, Paris, France

**Keywords:** Operational research, Community based tuberculosis care, Contribution, SORT IT

## Abstract

**Background:**

It is estimated that the standard, passive case finding (PCF) strategy for detecting cases of tuberculosis (TB) in Myanmar has not been successful: 26% of cases are missing. Therefore, alternative strategies, such as active case finding (ACF) by community volunteers, have been initiated since 2011. This study aimed to assess the contribution of a Community Based TB Care Programme (CBTC) by local non-government organizations (NGOs) to TB case finding in Myanmar over 4 years.

**Methods:**

This was a descriptive study using routine, monitoring data. Original data from the NGOs were sent to a central registry within the National TB Programme and data for this study were extracted from that database. Data from all 84 project townships in five regions and three states in Myanmar were used. The project was launched in 2011.

**Results:**

Over time, the number of presumptive TB cases that were referred decreased, except in the Yangon Region, although in some areas, the numbers fluctuated. At the same time, there was a trend for the proportion of cases treated, compared to those referred, that decreased over time (*P* = 0.051). Overall, among 84 townships, the contribution of CBTC to total case detection deceased from 6% to 4% over time (*P* < 0.001).

**Conclusions:**

Contrary to expectations and evidence from previous studies in other countries, a concerning reduction in TB case finding by local NGO volunteer networks in several areas in Myanmar was recorded over 4 years. This suggests that measures to support the volunteer network and improve its performance are needed. They may include discussion with local NGOs human resources personnel, incentives for the volunteers, closer supervision of volunteers and improved monitoring and evaluation tools.

**Electronic supplementary material:**

The online version of this article (doi:10.1186/s40249-017-0253-y) contains supplementary material, which is available to authorized users.

## Multilingual abstracts

Please see Additional file 1 for translations of the abstract into six official working languages of the United Nations.

**Fig. 1:**
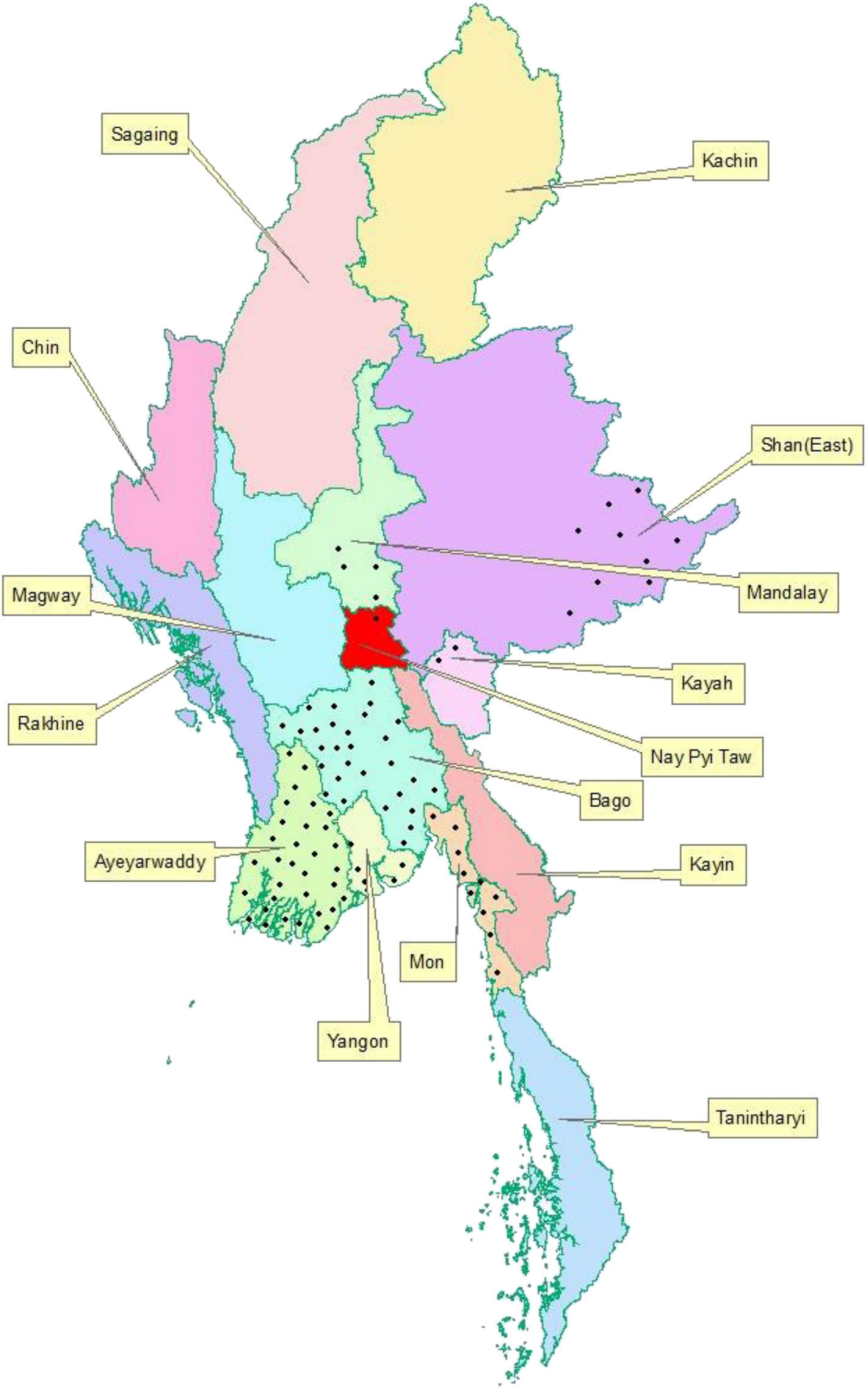
The location and coverage of CBTBC project 84 townships which were launched in 2011

## Background

Despite global efforts to control Tuberculosis (TB), the disease remains one of the world’s deadliest communicable diseases. According to the World Health Organization (WHO), an estimated 9.6 million people developed TB and 1.5 million died from the disease in 2014 [1]. Myanmar is one of the 30 high-burden TB countries with an estimated TB incidence and prevalence of 369 and 457 per 100 000 population, respectively, in 2014 (2). Despite national efforts to increase TB case finding, it is estimated that 26% of TB cases in Myanmar are still missing [2].

Case detection is one of the pillars of the TB control strategy recommended by the WHO. However, the standard passive case finding (PCF) strategy has not been successful in detecting all cases [3]. Globally, it is estimated that 37% of cases are undetected (1). Moreover, cases detected through traditional PCF experience long delays before diagnosis and treatment, thereby continuing the transmission of the disease in the community. One study in Myanmar showed a median delay of 60 days between onset of symptoms and diagnosis of TB [4], while another showed a median delay of 8 weeks between onset of symptoms and the start of treatment [5]. In addition, a large proportion of the population in Myanmar lives in hard-to-reach areas with limited access to health services because of difficulty in geographical access and security concerns [6].

Alternative strategies of case finding such as active case finding (ACF) by community volunteers have been shown to be effective [7]. In 2011 the National TB Programme (NTP) in Myanmar, in collaboration with four local NGOs, started Community Based TB Care (CBTC) in five regions and three states across the country. The programme involved screening individuals for TB symptoms in the community by community volunteers and referring those with presumptive TB to the township centre for diagnosis and treatment. In 2014, the 5^th^ WHO Joint Monitoring Mission pointed out that the number of local non-government organisations (NGOs) and community based organisations (CBOs) currently engaged in CBTC was small and recommended expansion of these activities [8].

Studies from Ethiopia have reported on the effectiveness and the role of ACF in increasing case finding rates [9, 10]. So far, there have been no studies on the effectiveness of the CBTC by local NGOs in Myanmar. Such an assessment is crucial to informed decision-making by the NTP and for better allocation of resources while expanding the programme.

Thus, the aim of this study was to assess the contribution of a CBTC programme by local NGOs to TB case-finding in five Regions and three States in Myanmar between 2011 and 2014. Specific objectives were to determine: 1. the number of presumptive TB cases referred by community volunteers to the township TB centre and the number and proportion of treated TB patients among presumptive TB cases over time 2. The number and proportion of treated TB cases reported by the CBTC programme out of the total TB cases notified in the townships where implemented CBTC in each regions and states over time.

## Methods

### Design

This was a descriptive study using routine programme monitoring data.

### General setting

Myanmar is a low-income country in South‐East Asia and is divided administratively into the Nay Pyi Taw Council Territory, seven states and seven regions. The population is approximately 51 million, 70% of whom reside in rural areas [11].

Health services in Myanmar are delivered through primary, secondary and tertiary health facilities. Tertiary health care services only exist in certain regions (Yangon, Mandalay, Nay Pyi Taw and Magwe). Other states and regions provide secondary and primary health care services. Primary health services are managed by the Department of Public Health, while secondary health services and tertiary health services are under the Department of Medical Services. In each region/state there are three to five districts where there is a district hospital and four to five township hospitals. In each township, there is one to two station hospitals, under which are four to five rural health centres. TB diagnosis and treatment services are provided in all townships. However, decentralization to station hospitals is underway. All TB services are provided free of charge across the country.

### Study sites

The study sites were chosen from five regions in Myanmar (Yangon, Mandalay, Nay Pyi Taw, Bago and Ayeyarwaddy) and three States (Eastern part of Shan, Mon, Kayah). They represented all areas where the CBTC programme by local NGOs was launched in 2011 and they covered a total of 84 townships in these regions and states. The primary objective of this programme was to involve the community in TB prevention and care activities.

### Description of CBTC activities by local NGOs

Local NGOs trained the community volunteers under the guidance of the NTP for their implementing townships. One volunteer covered 1–2 villages or wards; all villages or wards from each township were covered by a volunteer network. Their target population was presumptive TB cases in their respective townships.

CBTC included the following tasks and activities: (i) community health education; (ii) screening for TB symptoms of presumptive TB patients at community level (cough for 2 or more weeks, fever, loss of weight, night sweating and history of contact with smear-positive TB patients); (iii) referral of presumptive TB patients to township TB centres for diagnosis and treatment; (iv) providing directly observed treatment (DOT) service to TB patients; and (v) recording and reporting on the community-based TB care activities. All these tasks were performed by community health volunteers, except for diagnosis of TB, which took place in the township TB centres using smear microscopy and clinical assessment. The NTP endorsed “Guidelines for Community Based TB care to increase access to quality DOTS Service of presumptive TB patients” and all local NGOs and INGOs followed this guideline [6].

### Study population

All patients with presumptive and treated TB, detected through CBTC implemented by local NGOs between 2011 and 2014, were included in the study.

### Data variables, data collection and sources of data

Data variables included: (i) the number of presumptive TB cases referred and of these (ii) the number of treated TB cases each year, that were identified by local NGOs, and (iii) the number of total TB registered cases in each township. Data were sourced from the local CBTC NGOs’ monthly reports and the NTP’s annual reports. Local NGOs used standardized recording and reporting forms which were endorsed by the NTP. Original data from the NGOs were sent to a central registry within the NTP and data for this study were extracted from that database. Data were collected between July 2011 and December 2014.

### Analysis and statistics

Data were double-entered from paper-based extraction sheets into EpiData software (v3.1 EpiData Association, Odense, Denmark). A descriptive analysis was performed using simple proportions. The trends over time were assessed with the extended Mantel-Haenszel chi-square test for linear trend using the OpenEpi software [12] Statistical significance was set at 5%.

### Ethics

Ethics approval was obtained from the Ethics Committee of the Department of Medical Research (DMR), Yangon, Myanmar and from the Ethics Advisory Group of the International Union Against Tuberculosis and Lung Disease, Paris, France. Since only aggregated data were used and no names or other personal identifiers were included in any database, patient consent was not necessary.

## Results

The location and coverage of the CBTC projects’ 84 townships are shown in the Fig. 1. Table 1 shows the trends of presumptive and treated TB cases over time. Over 4 years, among 84 townships, the number of referred presumptive TB cases decreased, except in Yangon Region, although in some regions and states, the numbers fluctuated. At the same time, the proportion of cases treated, compared to those referred, decreased over time but the trend was not statistically significant (*P* = 0.051).

**Table 1:** Number of presumptive tuberculosis cases referred by community volunteers to township centres and proportion of those treated over time per region/state, Myanmar, 2011–2014

Region/state	Number of townships	Presumptive TB cases *n*				Treated TB patients *n* (%)				*P* value*
		2011^a^	2012	2013	2014	2011^a^	2012	2013	2014	
Ayeyarwaddy	26	727	4 015	2 023	1 865	270 (37)	1 560 (39)	577 (29)	522 (30)	<0.001
Bago	27	1 917	721	1 319	1 192	343 (18)	166 (23)	405 (31)	423 (36)	<0.001
Mandalay	4	671	1 112	766	854	133 (20)	188 (17)	191 (25)	179 (21)	0.049
Yangon	5	583	705	1 362	1 420	126 (22)	162 (23)	209 (15)	247 (17)	<0.001
Naypyitaw	1	19	10	18	11	5 (26)	2 (20)	3 (17)	0 (0)	0.060
Mon	10	1 167	1 079	379	932	166 (14)	178 (17)	40 (11)	85 (9)	<0.001
Kayah	2	83	118	562	206	8 (10)	25 (21)	128 (23)	58 (28)	0.001
Shan (East)	9	1 317	751	430	222	201 (15)	137 (18)	67 (16)	26 (12)	0.505
Total	84	6 484	8 511	6 859	6 702	1 252 (19)	2 418 (28)	1 620 (24)	1 540 (23)	0.051

*TB* tuberculosis

*Chi square test for linear trend for Treated TB patients

^a^Data were only available from last two quarters of 2011

Table 2 shows the proportion of referred and treated TB cases under the CBTC Programme compared to the total TB cases notified in the townships which had implemented CBTC. Decreasing proportions over time were seen in Bago, Naypyitaw, Mon and Shan (East) States, while those of Ayeyarwaddy Region and Kayah State increased. Overall, among 84 townships, the contribution of CBTC to total case detection decreased from 6% to 4% over time (*P* < 0.001).

**Table 2:** Number and proportion of tuberculosis patients referred and treated under the *Community Based TB Care Programme* by local non-governmental organizations out of the total TB cases notified in the townships where implemented CBTC in each region and state, Myanmar, 2011–2014

Region/state	Number of townships	Total registered TB cases *n*				TB patients referred and treated *n* (%)				*P* value*
		2011^a^	2012	2013	2014	2011^a^	2012	2013	2014	
Ayeyarwaddy	26	7 381	13 742	13 174	13 090	270 (4)	1 560 (11)	577 (4)	522 (4)	<0.001
Bago	27	5 943	12 359	12 699	13 111	343 (6)	166 (1)	405 (3)	423 (3)	0.012
Mandalay	4	898	1 596	1 362	1 130	133 (15)	188 (12)	191 (14)	179 (16)	0.130
Yangon	5	867	1 450	1 466	1 701	126 (15)	162 (11)	209 (14)	247 (15)	0.212
Naypyitaw	1	116	321	344	335	5 (4)	2 (1)	3 (1)	0 (0)	<0.001
Mon	10	3 291	6 563	7 010	6 421	166 (5)	178 (3)	40 (1)	85 (1)	<0.001
Kayah	2	215	612	608	641	8 (4)	25 (4)	128 (21)	58 (9)	<0.001
Shan (East)	9	1 183	1 862	1 668	1 757	201 (17)	137 (7)	67 (4)	26 (2)	<0.001
Total	84	19 894^a^	38 505	38 331	38 186	1 252 (6)^a^	2 418 (6)	1 620 (4)	1 540 (4)	<0.001

*TB* tuberculosis

*Chi square test for linear trend

^a^Data were only available from last two quarters of 2011

## Discussion

To our knowledge this was the first study of the contribution of the CBTC programme by local NGOs to TB case finding in Myanmar, and it revealed concerning results. The number of presumptive TB cases referred and the contribution of CBTC to total case detection actually dropped, overall, during the 4 years of the study. This is an important finding as it reveals issues that need to be addressed within the NTP.

One of the main objectives of the CBTC programme was to improve the contribution of TB case finding [6], in line with previous studies. One from Ethiopia showed that case detection rate was higher when trained community volunteers were employed in TB case finding [9]. Other studies from Cambodia and India reported the effectiveness and the role of Active Case Finding (ACF) in increasing case detection rates [13, 14]. A systematic review of the effectiveness of community based interventions showed a significant increase in TB case detection rates [15]. Thus, the findings from this study are in contrast to previous studies. Why is this so?

We suggest that several factors may be contributing. The local NGOs’ case finding procedure relies on volunteers to do the work. Volunteers do not get payment, so that while enthusiasm may be high at first, with time, it diminishes and the motivation to continue a significant workload is lost. In addition, there is a turnover among volunteers, but missing data make it difficult to quantify how much turnover there actually is. Finally, supervision of the volunteers may be weak and fail to motivate them. Linked to weak supervision may be inaccurate recording and reporting of data regarding notification of TB cases.

One study in Myanmar showed that activities of volunteers in TB case finding were limited because sputum transport charges from their villages to township were needed [16] to confirm the diagnosis. We also suggest that transportation fees for presumptive TB patients to attend the nearest diagnostic facility were not adequately covered and patients were unable to travel there. Although according to the NTP guidelines for community based TB care, sputum samples’ transportation costs to township TB centres could be provided to volunteers when the patient was unable to travel, we hypothesize that these fees were inadequately covered.

Another issue was missing data. Data were only available for referred presumptive TB cases and treated TB patients, whereas the number of presumptive TB cases who underwent sputum smear examination and those that were proven by bacteriological or clinical methods were not included in the reporting system of the CBTC programme. This lack of intermediate data prevented us from describing the complete diagnostic and treatment cascade and may have contributed to the unfavourable outcomes. This issue was illustrated by a large scale ACF study from India, where only 54% of referred patients underwent sputum smear examination and 8% were found to be smear positive [14].

There are some strengths to this study. Data from all 84 CBTC project townships, which were launched in 2011, were used and therefore there was no selection bias. The volunteers were all trained by local NGOs under the guidance of the NTP and following NTP-developed guidelines for this kind of work. All reported data was recorded and analysed centrally by the NTP. However, some limitations are recognised. The previously-mentioned lack of intermediate data was one. We have no record of the ongoing supervision of volunteers by the NGO staff, so this vital information is lacking. There was no verification of the reported data from the field at the NTP headquarters.

There are some important programmatic implications from this study. First, the results will be disseminated and discussed within the NTP and with the four local NGOs in a national seminar. Second, we suggest that further operational research, especially qualitative studies, should access the volunteers’ motivation and how to sustain volunteer participation. Another qualitative and quantitative mixed study should be conducted in Yangon Region because presumptive TB cases referred and those treated TB cases by CBTC were increasing while other regions and states showed decreasing trends. Third, there should be an assessment of the quality of supervision of the volunteers by NGOs and NTP staff. Forth, standardized incentives for volunteers should be considered given their rather extensive workload. In fact, they are carrying a major burden of health care for the NTP. Fifth, recording and reporting forms should be revised to obtain the missing data on diagnosis of TB cases and allow for the study of the complete diagnosis and treatment cascade. Finally, there should be a mechanism developed to assure that field data is accurately reported to headquarters.

## Conclusion

This study of CBTC in Myanmar, contrary to expectations and previous studies in other countries, revealed a concerning reduction in TB case finding by local NGO volunteer networks in several regions and states in Myanmar over 4 years. As a result, it points to a number of areas where the programme should be strengthened in the future in order for the NTP to realise the benefits of CBTC case finding.

## Additional file

Additional file 1: Multilingual abstracts in the six official working languages of the United Nations. (PDF 617 kb)

## Abbreviations

ACF: Active case finding
CBOs: Community Based Organisations
CBTC: Community Based Tuberculosis care
DMR: Department of Medical Research
DOT: Direct observation treatment
NGOs: Non-governmental Organizations
NTP: National Tuberculosis Programme
PCF: Passive case finding
TB: Tuberculosis
WHO: World Health Organization
