# Supplementary material for: The contribution of a non-governmental organisation’s Community Based Tuberculosis Care Programme to case finding in Myanmar: trend over time
Source: Infect Dis Poverty. 2017 Apr 3;6:51. doi: 10.1186/s40249-017-0253-y (PMC5376678; doi:10.1186/s40249-017-0253-y)
Supplement: Additional file 1: — Multilingual abstracts in the six official working languages of the United Nations. (PDF 617 kb) [file 40249_2017_253_MOESM1_ESM.pdf]

Translation of the abstract into the six official working languages of the United Nations

مساهمة برنامج الرعاية المجتمعية لمرض السل لمنظمة غير حكومية في نتائج دراسة في مينمار: الإتجاه مع مرور الوقت

هيت ميت وين مونغ، سو سو، بيتروس ايساكيديس، محمد كوجلي، انطوني ريد، غوين بين، هوا كو كو زاو، ساو ثين، سي ثو اونغ.

ملخص الدراسة
خلفية الموضوع: تشير التقديرات إلى أن معيار استراتيجية الحالات الكامنة للكشف عن حالات الإصابة بمرض السل في مينمار لم يكن ناجح: حيث أن 26% من الحالات في عداد المفقودين. ولذلك تم البدء باستخدام استراتيجيات بديلة مثل الحالات النشطة من قبل المتطوعين منذ عام 2011. تهدف هذه الدراسة إلى تقييم مساهمة برنامج الرعاية المجتمعية لمرض السل من قبل المنظمات المحلية الغير حكومية لحالات مرض السل في مينمار على مدى أربع سنوات.
منهجية البحث: تعد هذه الدراسة دراسة وصفية تمت من خلال استخدام بيانات المتابعة الروتينية. تم إرسال البيانات الأصلية المقدمة من المنظمات غير الحكومية إلى مركز التسجيل المركزي في إطار البرنامج الوطني لمرض السل وقد تم أستخراج البيانات التي تم استخدامها في هذه الدراسة من قاعدة البيانات تلك. واستخدمت بيانات من جميع بلدات المشروع في خمس مناطق وثلاث ولايات في مينمار. بدأ المشروع في عام 2011.
النتائج: انخفض عدد الحالات المحتملة لمرض السل التي تمت احالتها مع مرور الوقت ماعدا الحالات في منطقة يانغون على الرغم من أن الأرقام قد تقلبت في بعض المناطق. وفي الوقت نفسه كان هناك اتجاه لنسبة الحالات التي تم علاجها بالمقارنة مع الحالات التي تمت احالتها وقد انخفض هذا مع مرور الوقت (p=0.051). وبشكل عام من بين 84 بلدة مشاركة فإن مساهمة الرعاية المجتمعية لمرض السل إلى إجمالي الحالات المكتشفة انخفض من 6% إلى 4% مع مرور الوقت (P<0.001).
الخاتمة: على عكس التوقعات والأدلة من الدراسات السابقة في بلدان أخرى كان هناك انخفاض مقلق في اكتشاف حالات مرض السل من قبل شبكة المتطوعين في المنظمات الغير حكومية في مناطق عدة في مينمار على مدى أربع سنوات. ويشير هذا إلى ان هناك حاجة إلى اتخاذ تدابير لدعم شبكة المتطوعين وتحسين أداؤها. ويمكن أن تشمل هذه التدابير المناقشة مع موظفي الموارد البشرية في المنظمات الغير حكومية المحلية والحوافز للمتطوعين والإشراف على المتطوعين وتحسين أدوات الرصد والتقييم.

Translated from English version into Arabic by Randa82, through

TRANSLATORS
WITHOUT BORDERS

在缅甸以社区为基础的结核护理项目中非政府组织在结核病例发现中的贡献

Htet Myet Win Maung, Saw Saw, Petros Isaakidis, Mohammed Khogali, Anthony Reid, Nguyen Binh Hoa, Ko Ko Zaw, Saw Thein, Si Thu Aung

摘要
引言：据估计，在缅甸以标准被动病例发现策略检测肺结核病例是失败的，因为忽略了 26%的病例。因此，自 2011 年起采用了一些替换策略，比如依托社区志愿者的主动病例发现(ACF)策略。本研究的目的是评估 4 年来在以社区为基础的结核护理项目中，当地非政府组织(NGOs)在结核病例发现中的贡献。
方法：本研究是一项对常规监测数据的描述性研究。本研究的数据提取自被非政府组织提交到国家肺结核项目的中央注册中心的原始数据所构成的数据库。本项目始于 2011 年，数据包括缅甸 5 个地区和 3 个州的 84 个项目乡镇的全部数据。
结果：随着时间的推移，除仰光地区外，根据推定的肺结核病例数也有所降低，但是在一些地区，病例数有波动。同时，治疗病例的比例也有所下降。总体而言，84 个乡镇中，以社区为基础的结核护理项目在结核病例发现中的贡献，随着时间的推移从 6%降至 4% (P<0.001)。
结论：与先前在其他国家的研究证据和期望相反，在过去四年，在缅甸一些地区由当地非政府组织志愿者网络发现的结核病病例数呈下降趋势。这表明仍需采取措施支持志愿者网络并提升其效率。这些措施包括与当地非政府组织人力资源人员进行讨论、激励志愿者、加强监督志愿者，以及改善监测和评估的工具。

Translated from English version into Chinese by Xin-Yu Feng, edited by Pin Yang

CHINESE CENTER FOR DISEASE CONTROL AND PREVENTION
NATIONAL INSTITUTE OF PARASITIC DISEASES

Contribution du programme communautaire de lutte contre la tuberculose d'organisations non gouvernementales à la détection de cas au Myanmar: évolution au fil du temps

Htet Myet Win Maung, Saw, Petros Isaakidis, Mohammed Khogali, Anthony Reid, Nguyen Binh Hoa, Ko Zaw, Saw Thein, Si Thu Aung

RÉSUMÉ

Contexte: On estime que la stratégie normale de dépistage passif des cas de tuberculose (TB) au Myanmar n'a pas réussi: 26% des cas n'ont pas été détectés. Par conséquent, des stratégies alternatives, comme la recherche active de cas par des bénévoles communautaires, ont été lancées depuis 2011. Cette étude visait à évaluer la contribution d'un programme communautaire de lutte contre la tuberculose d'organisations non gouvernementales à la détection de cas au Myanmar.

Méthodes: Il s'agissait d'une étude descriptive utilisant des données de surveillance de routine. Les données provenant des ONG ont été envoyées à un registre central dans le cadre du programme national de lutte contre la tuberculose. Les données de la présente étude ont été extraites de cette même base de données. Les données utilisées proviennent de 84 cantons, dans cinq régions et trois États du Myanmar. Le projet a été lancé en 2011.

Résultats: Au fil du temps, le nombre de suspicions de tuberculose référées a diminué, excepté dans la région de Yangon, bien que dans certaines régions, le nombre ait fluctué. Parallèlement, la proportion de cas traités, comparés à ceux référés, a diminué avec le temps (P=0,051). Dans l'ensemble, parmi 84 cantons, la contribution du programme communautaire de lutte contre la tuberculose dans la détection totale des cas a diminué de 6% à 4% au fil du temps (P<0,001).

Conclusions: Contrairement aux attentes et aux résultats d'études antérieures menées dans d'autres pays, une réduction inquiétante des cas détectés de tuberculose par les réseaux locaux de volontaires d'ONG dans plusieurs régions du Myanmar a été enregistrée sur quatre ans. Cela suggère que des mesures de soutien au réseau de bénévoles et d'amélioration de leurs performances sont nécessaires. Ces mesures peuvent inclure des discussions avec le personnel des ressources humaines des ONG locales, des primes pour les bénévoles, une supervision plus étroite des bénévoles et l'utilisation d'outils de suivi et d'évaluation améliorés.

Translated from English version into French by Marion NOURRISSON

Практическое значение программы диагностики и лечения туберкулеза, основанной на местных ресурсах здравоохранения и поддерживаемой неправительственными организациями, для выявления случаев заболевания в Мьянме: тенденции изменений на протяжении определенного периода времени

Htet Myet Win Maung, Saw Saw, Petros Isaakidis, Mohammed Khogali, Anthony Reid, Nguyen Binh Hoa, Ko Ko Zaw, Saw Thein, Si Thu Aung

КРАТКИЙ ОБЗОР

Основная информация. Подсчитано, что стратегия пассивного выявления случаев (ПВС) заболевания туберкулезом (ТБ) при помощи стандартных методов в Мьянме не была успешной: не учтено 26% случаев. В связи с этим с 2011 года добровольцами общественных организаций инициированы альтернативные стратегии в виде активного выявления случаев (АВС). Целью этого исследования является оценка практического значения программы диагностики и лечения туберкулеза, основанной на местных ресурсах здравоохранения (Community Based TB Care Programme, CBTC) и поддерживаемой местными неправительственными организациями (local non-government organizations, NGOs), для выявления случаев заболевания ТБ в Мьянме за последние четыре года.

Методы. Это было описательное исследование с применением рутинных данных мониторига. Оригинальные данные из документов неправительственных организаций направляли в центральный реестр в рамках Национальной программы ТБ, а из этой базы данных извлекали информацию для этого исследования. Использовались данные всех 84 проектов, осуществляемых в небольших городках и селениях, которые расположены в пяти регионах и трех штатах Мьянмы. Этот проект был начат в 2011 году.

Результаты. За указанный период времени уменьшилось количество сообщений о предполагаемых случаях ТБ, за исключением округа Янгон (хотя показатели колебались в некоторых областях). За такой же период времени отмечена тенденция к снижению пропорции леченных случаев по сравнению с количеством сообщенных случаев (P=0.051). В целом на территории 84 небольших городков и селений практическое значение CBTC при полном выявлении случаев заболевания за указанный период времени уменьшилось с 6% до 4% (P<0.001).

Выводы: В противовес ожиданиям и данным, полученным из предыдущих исследований в других странах, за четыре года зафиксировано настораживающее снижение выявления случаев ТБ при помощи сети добровльцев местных неправительственных организаций в некоторых областях Мьянмы. Это дает основания предполагать, что нужны меры для поддержки сетей добровольцев и улучшения их

деятельности. Такие меры могут включать обсуждение вопросов с сотрудниками местных неправительственных организаций, которые контактируют с добровольцами, материальное стимулирование добровольцев, более тщательное наблюдение за их деятельностью и усовершенствование инструментов мониторинга и оценки.

Translated from English version into Russian by Ann Nosova, through

Contribución de los Programas Comunitarios para la Tuberculosis de organizaciones no gubernamentales para la identificación de casos en Myanmar: tendencias a lo largo del tiempo

Htet Myet Win Maung, Saw Saw, Petros Isaakidis, Mohammed Khogali, Anthony Reid, Nguyen Binh Hoa, Ko Ko Zaw, Saw Thein, Si Thu Aung

RESUMEN

Antecedentes: Se estima que la estrategia estándar para la identificación pasiva de casos para la detección de casos de tuberculosis (TB) en Myanmar no ha tenido éxito: se pierden el 26% de los casos. Por lo tanto, desde el año 2011 se han iniciado estrategias alternativas, como la identificación activa de casos por parte de voluntarios comunitarios. El presente estudio tiene como objetivo analizar la contribución de los Programas Comunitarios para la Tuberculosis de organizaciones no gubernamentales (ONG) locales para la identificación de casos de TB en Myanmar durante un período de cuatro años.
Métodos: El presente fue un estudio descriptivo que utilizó información de rutina y monitoreo. La información original de la ONG fue enviada a un registro central dentro del Programa Nacional para la TB y la información para el presente estudio se extrajo de dicha base de datos. Se utilizó información de 84 localidades proyecto en cinco regiones y tres estados de Myanmar. El proyecto se inició en el año 2011.
Resultados: Con el tiempo, la cantidad de casos presuntos de TB que fueron referidos disminuyó, excepto en la región de Yangon, aunque en algunas áreas las cifras fluctuaron. Al mismo tiempo, se observó con el correr del tiempo, una tendencia a la disminución en la proporción de casos tratados en comparación con aquellos referidos (P=0,051). En general, en las 84 localidades, la contribución del Programa Comunitario para la Tuberculosis al total de la detección de casos disminuyó con el correr del tiempo, del 6% al 4% (P<0,001).
Conclusiones: Contrario a lo que se esperaba y la evidencia de estudios previos en otros países, en el transcurso de cuatro años se registró una inquietante disminución en la identificación de casos de TB por parte de las redes voluntarias de ONGs en varias áreas de Myanmar. Esto sugiere que se necesitan medidas de apoyo para la red de voluntarios y también mejorar su desempeño. Las mismas pueden incluir charlas con el personal de recursos humanos de las ONGs locales, incentivos para los voluntarios, una supervisión más detallada de los voluntarios y el mejoramiento de las herramientas de monitoreo y evaluación.

Translated from English version into Spanish by Maria Alejandra Aguada, through
